# Prevalence and Associated Factors of Urinary Incontinence among Chinese Adolescents in Henan Province: A Cross-Sectional Survey

**DOI:** 10.3390/ijerph17176106

**Published:** 2020-08-21

**Authors:** Yan Luo, Ping Zou, Kai Wang, Zhenti Cui, Xiaomei Li, Jing Wang

**Affiliations:** 1 Faculty of Nursing, Health Science Center, Xi’an Jiaotong University, 76# Yanta West Road, Xi’an 710061, China; luoyan0904@xjtu.edu.cn (Y.L.); zhenti@stu.xjtu.edu.cn (Z.C.); roselee@xjtu.edu.cn (X.L.); 2 School of Nursing, Nipissing University, 750 Dundas West, Room 209, Toronto, ON M6J3S3, Canada; pingz@nipissingu.ca; 3 Department of Epidemiology and Biostatistics, School of Public Health, Tongji Medical College, Huazhong University of Science and Technology, 13# Hang Kong Road, Wuhan 430030, China; kay_wang@hust.edu.cn

**Keywords:** urinary incontinence, prevalence, associated factors, adolescents, China

## Abstract

Urinary incontinence is a common but understudied health problem in adolescents. This study aimed to investigate the prevalence of and associated factors for urinary incontinence in high-school-aged Chinese adolescents. A stratified two-stage cluster sampling procedure was adopted, yielding a sampling frame of 15,055 participants from 46 high schools in Henan province, China. Self-reported questionnaires were used to collect data. The urinary incontinence variable was assessed using the International Consultation of Incontinence Questionnaire-Short Form. The prevalence of urinary incontinence was 6.6%, with a female predominance (7.2% versus 6.0% in males; *p* < 0.05), and it increased with age, from 5.8% at 14–15 years to 12.3% at 19–20 years old (*p* < 0.001). The most common subtype of urinary incontinence was urgency urinary incontinence (4.4%), followed by stress urinary incontinence (1.7%) and mixed urinary incontinence (0.5%). Female sex, higher grades, more frequent sexual behavior, physical disease, chronic constipation, mental health problems, and residence in nonurban areas were significantly associated with higher odds of having urinary incontinence (*p* < 0.05). Public health programs, such as health education and school-based screening, should be established for early detection and appropriate management of urinary incontinence. Furthermore, individualized interventions targeting associated factors should be developed through collective efforts by adolescents, families, schools, and policymakers.

## 1. Introduction

Urinary incontinence (UI) is the involuntary leakage of urine, which may be defined as continuous or intermittent. According to the International Children’s Continence Society (ICCS), intermittent incontinence, including nocturnal enuresis and daytime incontinence, is diagnosed when the individual is aged 5 years or above and the condition lasts a duration of three months with a frequency of one or more episodes per month [1]. In contrast, the definition of adult UI developed by the International Continence Society (ICS) places a greater focus on defining signs and symptoms than a condition. Their definition includes three common types of UI: urgency UI, which is the complaint of involuntary loss of urine associated with urgency; stress UI, which is the complaint of involuntary leakage on effort or exertion, or on sneezing or coughing; and mixed UI, which is a combination of stress and urgency UI [2,3]. It is more appropriate to classify incontinence based on patient complaints than perceived anatomical descriptions. No specific classification is available for adolescents; however, the pediatric definitions can be useful in persistent and relapsing cases and the adult definitions might be applicable in new-onset cases [1,2].

Adolescence is a unique period in development during which certain tasks must be accomplished to successfully enter adulthood [4,5]. It can be subdivided into early (10–13 years), middle (14–16 years), and late adolescence (17–21 years) based on developmental tasks [4,5]. This is also a period when urine production and regulation change and differs between boys and girls [5]. In recent decades, many published studies have focused on the epidemiology of nocturnal enuresis in children and UI in adults, but incontinence in adolescents remains a neglected research topic [6,7,8]. Several large-scale epidemiologic surveys of nocturnal enuresis have been conducted in Korea, the USA, Turkey, and other countries, revealing that the prevalence of nocturnal enuresis differs with race and ethnicity [1,9,10]. The overall prevalence of daytime incontinence and nocturnal enuresis among adolescents is reported to be 1.0–1.8% and 0.5–2.6%, respectively [6]. In general, the prevalence of nocturnal enuresis in Asia is lower than that reported in Western countries [1,9,10]. The most recent large-scale epidemiological survey of primary nocturnal enuresis in mainland China was done during 2003–2004 among 10,088 children and adolescents aged 5–18 years old. An overall prevalence of 4.1% (5.7% in children and 1.5% in adolescents) was reported [11]. In a school-based Hong Kong study of 16,512 children and adolescents aged 5–19 years, the rate of primary nocturnal enuresis was 1.8% for the 13–19 years old group [12]. However, both surveys focused only on primary nocturnal enuresis and the prevalence of other subtypes of incontinence is unclear. Few large-scale epidemiological surveys of UI among adolescents using the current ICS definitions have been conducted in China or worldwide.

Management of adolescents with UI is superimposed considering the rapid physical, psychological, and developmental changes that occur in this population. Adolescents who carry UI from adolescence to adulthood can be considered similar to those with a chronic illness, in whom development, body image, and socialization are likely to be disrupted [13,14]. As effective treatments for UI are available, public awareness and early detection of UI among adolescents is critical. Currently, care for adolescents is mostly provided by either pediatric or adult medical services [15]. Addressing the prevalence and predictors of UI in adolescents is essential to inform health services and individualized interventions development for symptom alleviation and comorbidity prevention. Therefore, the aims of the present study were to describe the presentation of UI as well as associated factors among high-school-aged adolescents in China.

## 2. Materials and Methods

### 2.1. Study Design

From June to August 2018, a school-based epidemiological survey was performed in Henan province, which is located in the central area of China and features the largest number of high school students among the hinterland provinces.

### 2.2. Ethical Considerations

This study was approved by the Ethics Committee of Health Science Centre, Xi’an Jiaotong University, and the local schools that participated in this study (project number: 2018-296). All participants and their parents were informed in detail about the study, potential risks and benefits, and participants’ roles and signed an informed consent before data collection. Participation was voluntary and anonymity was guaranteed.

### 2.3. Sample and Setting

In China, at the age of 6 years, children enter the first grade of primary education, which lasts 6 years. After primary school and 3 years of middle school, students attend high school for 3 years before taking the national university entrance examination. If they need to retake this examination, they may take another year of high school with the third-year students. So, some fourth-year students were also included in this study.

At the time that this survey began, more than 2.0 million adolescents were studying at 852 high schools in Henan province, with an average of 2465 students in each school. As the main aim of this study was to evaluate the UI prevalence among adolescents, sample size was calculated by N = $Z_{\alpha /2}^{2}$ P $\left(1-P\right)/d^{2}$. With the maximum value of 0.25 for P (1 − P), α = 0.05 (representing I type error, $Z_{\alpha /2}$ = 1.96 accordingly), and 1% absolute error d, a sample size of 9604 would be sufficient. Considering a 30% non-respondent rate or missing data, the sample size was estimated to be 13,720 adolescents. Assuming that 300 students (100 students in each grade) were selected from each high school, 46 high schools would be needed.

A stratified two-stage cluster sampling procedure was used and participants were selected from 46 high schools located in all 17 provincial cities in Henan province. In Stage 1, 46 high schools were selected with probability proportional to enrollment size. In Stage 2, grades were considered as strata and 2–3 classes in each grade of the 46 selected high schools were randomly selected as clusters based on the class size. All students in the selected classes were invited to participate in the study by their head teachers and individuals willing to take part were provided detailed written information about data collection procedures and instructions. All participants completed data collection during self-study classes in the presence of the principal investigator.

### 2.4. Instrumentation

#### 2.4.1. Demographic Information

The demographic information form was developed based on literature about the factors associated with adolescents’ UI, including demographics (age, sex, height, weight, grade, ethnicity, residence on campus or not), physiopathology (smoking, alcohol consumption, sexual behavior, frequency of sexual behavior in the last month, gaming addiction), medical history (physical disease, chronic constipation defined according to the Rome IV criteria [16]), and family factors (inhabitation, family type, parents’ education level). Age was subdivided into two groups: middle adolescence (14–16 years) and late adolescence (17–21 years) based on developmental tasks [4,5]. Body mass index (BMI) was calculated and categorized into three groups: underweight, normal weight, and overweight [17].

#### 2.4.2. International Consultation on Incontinence Questionnaire-Short Form (ICIQ-SF)

The UI variable was investigated using the Chinese Version of ICIQ-SF [18], which was strongly recommended by the Association of the Scientific Medical Societies’ current guidelines [19]. The ICIQ-SF is applicable to new-onset cases in patients of all ages. Participants were asked whether they experienced urinary leakage during the past four weeks in connection with “experiencing incontinence before she/he could reach a toilet, during sleep, after a toilet visit, without reason and constantly”, which indicated urgency UI, or “coughing and/or sneezing, or incontinence in addition to physical activity”, which indicated stress UI. The participant who reported a combination of urgency and stress symptoms was identified as having mixed UI. Nocturnal enuresis is defined as loss of urine during sleep [3], which is included in the urgency UI according to ICIQ-SF [18]. The ICIQ-SF consisted of four items that assessed (1) the frequency; (2) severity; (3) impact of UI on daily life (0–10 points: 0 = none, 1–3 = mild, 4–6 = moderate, 7–9 = severe, 10 = very severe); and (4) situation or causes leading to UI. The overall ICIQ-SF scores were calculated by the sum of items 1 to 3, with a total score of 1 or above indicating incontinence.

#### 2.4.3. Mental Health Inventory of Middle-School Students (MMHI-60)

The mental health variable was investigated using the MMHI-60, a self-administered screening test designed to assess general mental health among Chinese middle and high school students [20]. The MMHI-60 consists of 60 items, each requiring the respondent to indicate on a five-point scale whether they have recently experienced a particular symptom or type of behavior. Examples of the questions are “Have you recently been feeling nervous and strung-up all the time; frequently thought of committing suicide; and feeling unable to solve problems”. Mental health status is divided into five categories ranging from normal to very serious, with cutoff points of 2 (mild), 3 (moderate), 4 (serious), and 5 (very serious). The test-retest reliability of MMHI-60 reported by Wang et al. was 0.873 [20]. The Cronbach’s alpha in this study is 0.967.

### 2.5. Data Analysis

Data were entered in duplicate using EpiData 3.1 software (the Epidata Association, Odense, Denmark) and then were exported to R software (version 3.6.2, R Foundation, Florida, USA) for analysis. Continuous variables were expressed as the mean ± SD and were analyzed by two independent sample *t*-tests. Categorical or ordinal variables were expressed as absolute (n) and relative (%) frequencies. Estimates of the prevalence of UI with 95% confidence intervals were calculated separately for the overall population and for subgroups stratified by age and sex. The prevalence differences among subgroups were compared with Pearson’s χ^2^-test. Univariate logistic regression was performed to select possible associated factors for UI with *p* values of less than 0.05. A multivariate logistic regression model with a backward variable selection method was employed to identify the net effect of factors contributing to UI. Variables significantly associated with UI in univariate analysis and reported by previous studies were entered as independent variables in the multivariate regression analysis. A two-sided *p* value less than 0.05 was considered statistically significant.

## 3. Results

**Figure 1:**
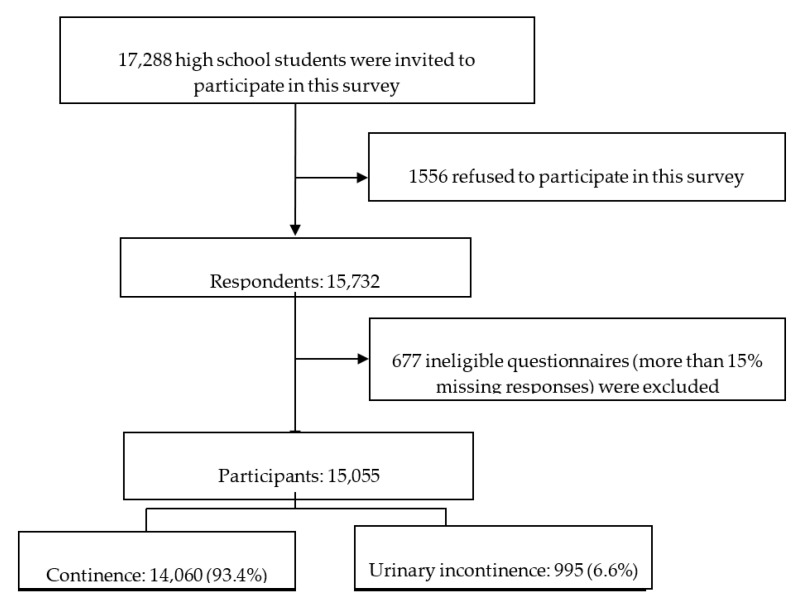
Flowchart of participants through this study.

In total, 15,732 (91.0%) students from 46 high schools participated in this survey. Respondents who provided incomplete data were excluded. Overall, 15,055 students (7514 boys and 7541 girls) were recruited in the present study (see Figure 1).

### 3.1. Demographic Information

Major characteristics of all participants are presented in Table 1. The mean age of participants was 16.73 years old (SD = 0.88, ranging from 14 to 20). Approximately half of the participants were girls (50.1%) and came from rural areas (55.9%). The majority of participants were Han Chinese (97.5%), came from non-single parent families (93.4%), and lived on campus (73.1%). In total, 722 (4.8%) participants consumed cigarettes and 13.0% consumed alcohol. Further, 674 (4.5%) participants had been sexually active and 6.4% of the participants had at least one kind of physical disease.

### 3.2. Prevalence of UI

The overall prevalence rates of UI, urgency UI, stress UI, and mixed UI were 6.6% (995/15,055), 4.4% (660/15,055), 1.7% (254/15,055), and 0.5% (81/15,055), respectively. The prevalence of UI increased with age, ranging from 5.8% at 14–15 years old to 12.3% at 19–20 years old (χ^2^ = 4.543, *p* < 0.001; Table 2). There was an increasing trend in UI prevalence with age in both sexes (both *p* < 0.05; Table 2).

**Figure 2:**
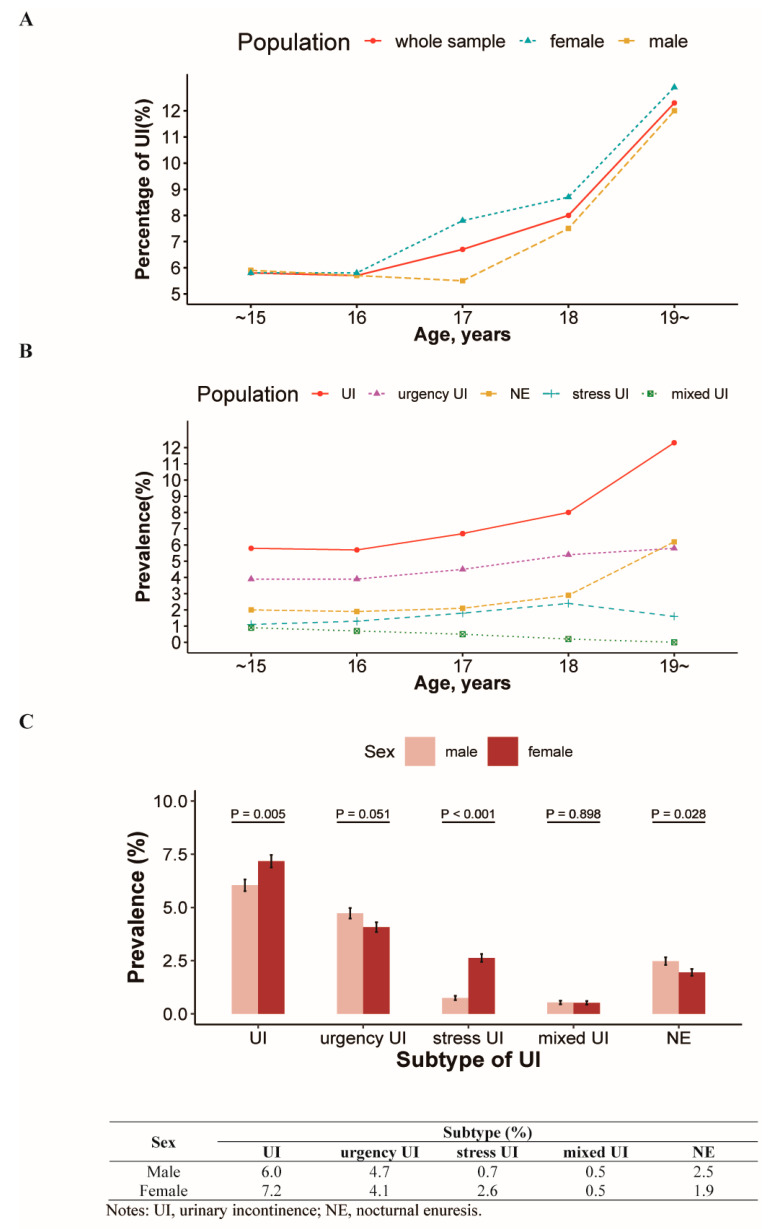
The prevalence of UI and UI subtype by age group and sex. (**A**) Prevalence of UI by age group; (**B**) Prevalence of UI subtype by age group; (**C**) Prevalence of UI subtype by sex.

The prevalence of UI and UI subtypes by age and sex is shown in Figure 2. Incontinence was found to occur more frequently in girls than in boys (7.2% versus 6.0%, *p* < 0.05). Of the UI subtypes, the prevalence of urgency UI was the highest in each age group. Girls had a significantly higher prevalence of stress UI compared to boys (2.6% versus 0.7%, *p* < 0.001) as well as a lower prevalence of nocturnal enuresis (1.9% versus 2.5%, *p* < 0.05). There were no sex differences in the prevalence of mixed UI or urgency UI.

Among all UI cases, the prevalence of UI symptoms was as follows: daytime frequency, 15.5% (154/995, not shown in the table) and leakage of only a small volume, 87.1% (867/995). Incontinence most commonly occurred during sleep (2.2%), followed by before reaching the toilet (1.8%) and when coughing or sneezing (1.5%; Table 3). Regarding the impact of UI on daily life, 79.9% (795/995), 9.3% (93/995), 2.0% (20/995), and 8.8% (87/995) of participants reported mild, moderate, serious, severe, and very severe impacts, respectively (Table 3).

### 3.3. Associated Factors of UI

The univariate analysis of demographic variables is presented in Table 4. UI was more likely to be reported among students who were older, female, in higher grades, lived on campus, consumed alcohol or cigarettes, engaged in sexual behavior, had a physical disease, experienced gastropathy, had chronic constipation, reported game addiction, had mental health problems, or had a single parent (*p* < 0.05). Students who lived in urban areas or had a parent with a bachelor’s degree showed lower odds of UI (*p* < 0.05).

In terms of the UI subtypes, older age, female sex, overweight, having a family living in a rural area, higher grades, residence on campus, more frequent sexual behavior, having a physical disease, experiencing chronic constipation, reporting mental health problems, and low paternal education (a maximum of the lower-secondary level) were associated with higher odds of having stress UI (*p* < 0.05). Older age, living in nonurban area, higher grades, residence on campus, cigarette or alcohol consumption, engagement in sexual behavior, gaming addiction, having a physical disease, experiencing gastropathy, having chronic constipation, reporting mental health problems, having a single-parent family, and having a father with a PhD degree were found to be correlated with higher odds of having urgency UI and nocturnal enuresis, while female sex and Han ethnicity were correlated with lower odds of having nocturnal enuresis (*p* < 0.05). Mixed UI was more likely to be reported among students who consumed alcohol or cigarettes, engaged in sexual behavior, had a physical disease, experienced gastropathy, had chronic constipation, and reported mental health problems, while was less likely among students in the older age group (*p* < 0.05; Table 4).

The results of multivariate analysis are presented in Table 5. Female sex, higher grades, more frequent sexual behavior, physical disease, chronic constipation, and mental health problems were associated with higher odds of having UI, while living in an urban area or having a father with a bachelor’s degree were correlated with lower odds (*p* < 0.05). Serious mental health problems were the strongest associated factor for UI (OR = 4.57, 95% CI = 2.27–9.27), followed by more frequent sexual behavior in the last month (>five times per week) (OR = 4.48, 95% CI = 3.07–6.54) and chronic constipation (OR = 2.03, 95% CI = 1.72–2.40).

In multivariate analyses (see Table 5), higher grades, physical disease, and mental health problems were common factors associated with all UI subtypes and other detected key associated factors for stress UI were female sex, more frequent sexual behavior, and low paternal education (a maximum of the lower-secondary level); and those for both urgency UI and nocturnal enuresis were living in nonurban areas, alcohol consumption, more frequent sexual behavior, and chronic constipation. Adolescents with a father with a PhD degree were significantly associated with higher odds of having nocturnal enuresis. The odds of having mixed UI was greater in adolescents who were smoking (OR = 2.24, 95% CI = 1.15–4.35) and had chronic constipation (OR = 3.64, 95% CI = 2.25–5.9), while lower in adolescents who were older (OR = 0.48, 95% CI = 0.29–0.8).

## 4. Discussion

There are several important findings of this study. First, our data indicate that 6.6% of Chinese adolescents aged 14–20 years old had UI as defined by the ICS. Second, the prevalence of UI was significantly higher in girls and increased with age. Finally, mental health problems were strongly associated with UI, providing a new path for development of schools’ public health services to improve adolescent’s mental health.

An epidemiological study by Bardino et al. [21], which also investigated the UI variable using ICIQ-SF among 1936 nulliparous 15- to 25-year-old women, reported a prevalence of UI at 14.2% among women aged 15 to 21 years. A possible reason for differences in the prevalence rates might be the younger age of participants in this study, consistent with previous studies reporting that advancing age is an associated factor for UI, especially stress UI [22]. Therefore, when comparing the prevalence of UI between studies, consideration should be given to the age of the respondents as well as to the methods of data collection. In our study, the odds of having UI was 1.27 times higher in girls than in boys and sex differences were found in the distribution of different UI subtypes. Nocturnal enuresis was more common among boys than girls, as was observed by other studies in China [11,12]. Since general continence is clearly linked to developmental maturity, perhaps females experience fewer problems in this regard because they mature earlier than males on average. However, stress UI was significantly more frequent in girls than in boys. The multi-factorial form of stress UI observed in females is different from that observed in males. Anatomic changes in the pelvic organs due to obesity, chronic cough, constipation, parturition, or menopause are important factors associated with female incontinence [22]. These sex-specific factors may differentially predispose male and female patients to UI, indicating a need for individualized management and coping strategies.

Findings of this study showed that the prevalence of UI is significantly increased with age, consistent with other studies, which found that the prevalence of daytime UI was greater among adolescents with advancing age [11,12]. Yeung et al. [12] found that daytime incontinence symptoms affected 29.2% of 10–19-year-old adolescents with nocturnal enuresis, but only 13.6% of 5–10-year-old children. One possible reason may be that many risky behaviors, such as smoking, binge drinking, and early sexual debut, begin in or are observed throughout adolescent development, especially middle adolescence (14–16 years) [4]. The Youth Risk Behavior Surveys, which were conducted among American high school students, showed that 8.8% of the respondents were current smokers, 16.5% endorsed binge drinking, and 39.5% have had sexual intercourse [23]. These behaviors have been found to be significantly associated with the development of UI in previous studies [1,24]. Similar correlations were also found in our study. Although the overall prevalence rate of UI increased with age, we found an inverse association of age group and mixed UI. In our study, the odds of having mixed UI was lower in late adolescents (17–20 years) than that in middle adolescents (14–16 years). Risk factors appear to differ in females and males due to anatomic differences and UI pathophysiology [22], which is also verified by the sex difference in the prevalence of stress UI and nocturnal enuresis in our study. Considering sex difference in risky behaviors between middle and late adolescence [1], the patient population of stress and urgency UI may not be the same, resulting in a decreased rate of the combination of stress and urgency UI in the same population.

For both boys and girls, the odds of UI and subtypes were strongly associated with mental health problems, which supports previous studies suggesting that psychological comorbidities are more common among individuals with UI in all age groups [14,25]. It is believed that the association between mental health problems and incontinence is bidirectional. Mental health problems are found to be a consequence of UI; embarrassment, distress, and loss of self-esteem are often reported by those affected by the condition [14]. In addition, mental health problems may precede UI symptoms, especially nocturnal enuresis, due to the comorbidities associated with mental health problems (i.e., delayed bladder control, general development delay, and behavior problems) [13]. Further prospective longitudinal studies are needed to examine whether adolescents’ incontinence is an associated factor for later mental health problems. Furthermore, we found that UI and mental health problems shared common associated factors; for example, adolescents with UI or mental health problems both tended to be living in nonurban areas, have physical disease, and have unhealthy behaviors (such as smoking, alcohol consumption, and engagement in sexual behavior), in line with other studies [26,27]. Further research is warranted to identify the proxy mechanisms between UI and mental health problems. In addition, psychological symptoms and disorders need to be considered in the assessment and treatment of UI and education programs about UI symptoms and development of the UI care system might facilitate early detection and appropriate management of adolescent UI, which may be beneficial for preventing mental health problems among this group [28].

In our study, frequency of sexual behavior was strongly associated with UI and subtypes, including stress UI, urgency UI, and nocturnal enuresis. The odds of having UI for adolescents who engaged in sexual activity more than 5 times per week in the last month were 4.48 times that of adolescents who did not engage in sexual activity. These observations are consistent with a study of nulliparous women aged 16–30 years in Australia, which found that UI was associated with sexual activity, either current or in the past [27]. Currently, most studies focus on the impact of UI on sexual function, but the mechanism through which sexual behavior increases the prevalence of UI is rarely investigated [25]. One possible reason for this association may be that unsanitary sexual intercourse is significantly associated with urinary tract infection, which is an important risk factor for UI [29,30]. The significant association of sexual activity at a currently socially unaccepted, young age with UI might open a new avenue for preventing or postponing the onset of UI. Our results also suggest the importance of improving sex education in high schools through the combined efforts of families and schools.

In agreement with previous studies, this study found that physical diseases, chronic constipation, smoking, and alcohol consumption were significantly associated with UI [1,24]. A similar correlation between UI and medical comorbidities is reported by several studies [1]. Adolescents with physical illnesses, such as obesity, metabolic syndrome, diabetes mellitus, and cystic fibrosis, had a higher rate of UI than the general population [1,24]. Stress UI can be elicited by coughing and sneezing [22]. Chronic constipation may weaken pelvic floor muscles and increase intra-abdominal pressure due to damage to the pelvic floor tissue and weakening of ligaments from constant straining [19]. Constipation may also increase the frequency of UI by placing pressure on the bladder. Incontinence prophylaxis programs with healthy lifestyle elements should be established for adolescents. Such programs could involve discontinuation of smoking to help eliminate chronic cough, appropriate and moderate consumption of alcohol to relieve the possible diuretic effects, and control of constipation by avoiding excessive pressure during defecation, which predisposes individuals to stress UI [19,31].

Familial factors that were associated with UI in this study included inhabitation and paternal education level. We found that nonurban types of adolescents had higher instances of self-reported UI, especially nocturnal enuresis, than their counterparts who lived in urban areas, which is in keeping with the previous study among Chinese children and adolescents [11]. This geographic difference might be related to sanitation, health literacy, personal hygiene, and healthcare resources. In general, the public health conditions and living environment in urban areas are relatively better than those in nonurban areas in China [32]. Additionally, we found adolescents of fathers with low education (a maximum of the lower-secondary level) had higher odds of having stress UI compared to adolescents of fathers with bachelor’s degrees. However, adolescents of fathers with a PhD degree had higher odds of reporting nocturnal enuresis than those whose fathers had a bachelor’s degrees. Paternal education level was categorized into three groups: high school or below, technical college, and university or above in previous studies and higher paternal educational level was identified to be a protective factor for nocturnal enuresis and seeking active treatment in children and adolescents [33,34]. Research evidence on the association of paternal education and UI were too limited to draw any conclusions. Longitudinal population-based studies are encouraged in the future.

This cross-sectional study has significant advantages over other studies in terms of its large sample, rigorous criteria for UI assessment, and high response rate. However, this study has several limitations. Despite the large sample size, this study focused on a sample from one province and the findings cannot be generalized to the whole adolescent population in China. In addition, due to the nature of a cross-sectional design, the inference of causal relationships cannot be addressed.

## 5. Conclusions

This study suggests that UI occurs in both sexes, with a female predominance and an increased prevalence with age. Mental health problems and sexual behavior were associated with a higher prevalence of UI, with more than four-fold increased odds. Habits related to UI should be reconsidered to prevent symptoms and cures to improve UI should be developed. More extensive studies are required to demonstrate the relationship between UI and mental health among adolescents.

## Figures and Tables

**Table 1:** Prevalence of UI and univariate analysis of risk factors.

Related Factors		Total	Non-UI	UI	*p* Value
Age (years)	14–16	5881 (39.1)	5543 (94.3)	338 (5.7)	0.001
	17–20	9174 (60.9)	8517(92.8)	657 (7.2)	
Sex	Boys	7514 (49.9)	7060 (94.0)	454 (6.0)	0.005
	Girls	7541 (50.1)	7000 (92.8)	541 (7.2)	
BMI	Underweight	1346 (9.1)	1257 (93.4)	89 (6.6)	0.671
	Normal weight	12,294 (82.7)	11,493 (93.5)	801 (6.5)	
	Overweight	1226 (8.2)	1138 (92.8)	88 (7.2)	
Ethnicity	Han	14,679 (97.5)	13,710 (93.4)	969 (6.6)	0.809
	Minority	376 (2.5)	350 (93.1)	26 (6.9)	
Inhabitation	Urban	5984 (39.7)	5675 (94.8)	309 (5.2)	<0.001
	Rural	8411 (55.9)	7788 (92.6)	623 (7.4)	
	Rural-urban continuum	660 (4.4)	597 (90.5)	63 (9.5)	
Grade	First year	5279 (35.1)	4989 (94.5)	290 (5.5)	<0.001
	Second year	4009 (26.6)	3759 (93.8)	250 (6.2)	
	Third year	5620 (37.3)	5182 (92.2)	438 (7.8)	
	Fourth year	147 (1.0)	130 (88.4)	17 (11.6)	
Residence on campus	No	4047 (26.9)	3836 (94.8)	211 (5.2)	<0.001
	Yes	11,008 (73.1)	10,224 (92.9)	784 (7.1)	
Smoking	No	14,333 (95.2)	13,420 (93.6)	913 (6.4)	<0.001
	Yes	722 (4.8)	640 (88.6)	82 (11.4)	
Alcohol consumption	No	13,091 (87.0)	12,281 (93.8)	810 (6.2)	<0.001
	Yes	1964 (13.0)	1779 (90.6)	185 (9.4)	
Sexual behavior	Ever active	674 (4.5)	572 (93.8)	102 (6.2)	<0.001
	Never active	14,381 (95.5)	13,488 (84.9)	893 (15.1)	
Frequency of sexual behavior in the last month	0	14,447 (96.0)	13,554 (93.8)	893 (6.2)	<0.001
	1/w	339 (2.3)	299 (88.2)	40 (11.8)	
	2–3/w	65 (0.4)	59 (90.8)	6 (9.2)	
	4–5/w	41 (0.3)	32 (78.0)	9 (22.0)	
	>5/w	163 (1.1)	116 (71.2)	47 (28.8)	
Physical disease	No	14,086 (93.6)	13,225 (93.9)	861 (6.1)	<0.001
	Yes	969 (6.4)	835 (86.2)	134 (13.8)	
Gastropathy	No	14,894 (98.9)	13,923 (93.5)	971 (6.5)	<0.001
	Yes	161 (1.1)	137 (85.1)	24 (14.9)	
Coryza	No	14,944 (99.3)	13,955 (93.4)	989 (6.6)	0.608
	Yes	111 (0.7)	105 (94.6)	6 (5.4)	
Chronic constipation	No	13,471 (89.5)	12,704 (94.3)	767 (5.7)	<0.001
	Yes	1584 (10.5)	1356 (85.6)	228 (14.4)	
Game addiction	No	13,982 (92.9)	13,092 (93.6)	890 (6.4)	<0.001
	Yes	1073 (7.1)	968 (90.2)	105 (9.8)	
Single parent	No	14,061 (93.4)	13,149 (93.5)	912 (6.5)	0.022
	Yes	994 (6.6)	911 (91.6)	83 (8.4)	
Father’s education level	High school & below	10,790 (71.7)	10,052 (93.2)	738 (6.8)	<0.001
	Junior college	2208 (14.7)	2077 (94.1)	131 (5.9)	
	Bachelor	1700 (11.3)	1616 (95.1)	84 (4.9)	
	Master	140 (0.9)	130 (92.9)	10 (7.1)	
	PhD	217 (1.4)	185 (85.3)	32 (14.7)	
Mother’s education level	High school & below	11,436 (76.0)	10,668 (93.3)	768 (6.7)	<0.001
	Junior college	1748 (11.6)	1634 (93.5)	114 (6.5)	
	Bachelor	1569 (10.4)	1497 (95.4)	72 (4.6)	
	Master	101 (0.7)	89 (88.1)	12 (11.9)	
	PhD	201 (1.3)	172 (85.6)	29 (14.4)	
MMHI-60	Normal	8768 (58.2)	8387 (95.7)	381 (4.3)	<0.001
	Mild	5538 (36.8)	5049 (91.2)	489 (8.8)	
	Moderate	702 (4.7)	592 (84.3)	110 (15.7)	
	Serious and very serious	47 (0.3)	32 (68.1)	15 (31.9)	

Abbreviations: UI, urinary incontinence; BMI, body mass index; MMHI-60, Mental Health Inventory of Middle-School Students.

**Table 2:** Prevalence of UI in different age groups.

Age (Years)	Boys			Girls			Total			*p* Value *
	N (%)	UI	Rate (95%CI), %	N (%)	UI	Rate (95%CI), %	N (%)	UI	Rate (95%CI), %	
14–15	473 (6.3)	28	5.9 (3.7, 8.1)	538 (7.1)	31	5.8 (3.8, 7.8)	1011 (6.7)	59	5.8 (4.4, 7.2)	0.915
16	2293 (30.5)	130	5.7 (4.7, 6.7)	2577 (34.2)	149	5.8 (4.8, 6.8)	4870 (32.4)	279	5.7 (5.1, 6.3)	0.866
17	3294 (43.8)	181	5.5 (4.7, 6.3)	3288 (43.6)	258	7.8 (6.8, 8.8)	6582 (43.7)	439	6.7 (6.1, 7.3)	<0.001
18	1312 (17.5)	98	7.5 (6.1, 8.9)	1037 (13.8)	90	8.7 (6.9, 10.5)	2349 (15.6)	188	8.0 (6.8, 9.2)	0.283
19–20	142 (1.9)	17	12.0 (6.7, 17.3)	101 (1.3)	13	12.9 (6.4, 19.4)	243 (1.6)	30	12.3 (8.2,16.4)	0.834
Total	7514	454	6.0 (5.4, 6.6) ^#^	7541	541	7.2 (6.6, 7.8) ^§^	15,055	995	6.6 (6.2, 7.0)	0.005

Abbreviations: UI, urinary incontinence; CI: confidence interval. * Asymp. Sig. with Chi-Square test on the prevalence rate of UI between boys and girls in different age groups. ^#^ *p* < 0.05 with Cochran-Armitage Trend (χ^2^ = 5.515) on the prevalence rate of UI in the different age groups of Chinese boys. ^§^ *p* < 0.01 with Cochran-Armitage Trend (χ^2^ = 4.124) on the prevalence rate of UI in the different age groups of Chinese girls.

**Table 3:** Severity of UI.

Characteristics		N (%)
Frequency of urinary leakage	Never	14,060 (93.4)
	Approximately once per week or less often	733 (4.9)
	Two or three times per week	108 (0.7)
	Approximately once per day	50 (0.3)
	Several times per day	40 (0.3)
	All the time	64 (0.4)
Amount of usual leakage	A small amount	867 (87.1)
	A moderate amount	58 (5.8)
	A large amount	70 (7.1)
Impact of UI on daily life	Mild	795 (79.9)
	Moderate	93 (9.3)
	Severe	20 (2.0)
	Very severe	87 (8.8)
The situation or causes leading to UI	Never—urine does not leak	14,060 (93.4)
	Leaks before you can get to the toilet	273 (1.8)
	Leaks when you cough or sneeze	225 (1.5)
	Leaks when you are asleep	333 (2.2)
	Leaks when you are physically active/exercising	153 (1.0)
	Leaks when you have finished urinating and are dressed	220 (1.5)
	Leaks for no obvious reason	211 (1.4)
	Leaks all the time	83 (0.6)

Abbreviations: UI, urinary incontinence.

**Table 4:** Univariate analysis of factors associated with UI and subtypes.

Related Factors		UI OR (95%CI)	Stress UI OR (95%CI)	Urgency UI OR (95%CI)	Mixed UI OR (95%CI)	NE OR (95%CI)
Age (years)	14–16 (ref.)					
	17–20	1.27 (1.10–1.45)	1.63 (1.24–2.15)	1.26 (1.07–1.49)	0.56 (0.36–0.88)	1.25 (1.00–1.58)
Sex	Boys (ref.)					
	Girls	1.20 (1.06–1.37)	3.59 (2.66–4.84)	0.87 (0.74–1.01)	0.97 (0.62–1.51)	0.78 (0.63–0.97)
BMI	Underweight (ref.)					
	Normal weight	0.98 (0.78–1.23)	1.66 (0.96–2.86)	0.98 (0.74–1.29)	0.92 (0.44–1.91)	0.88 (0.61–1.26)
	Overweight	1.09 (0.80–1.48)	2.06 (1.07–3.97)	1.12 (0.78–1.62)	0.55 (0.16–1.82)	0.64 (0.37–1.12)
Ethnicity	Han (ref.)					
	Minority	1.05 (0.70–1.57)	0.31 (0.08–1.23)	1.44 (0.94–2.21)	0.00 (0.00-Inf)	1.74 (1.01–3.00)
Inhabitation	Urban (ref.)					
	Rural	1.47 (1.28–1.69)	1.47 (1.12–1.92)	1.46 (1.23–1.73)	1.00 (0.62–1.59)	1.68 (1.31–2.15)
	Rural-urban continuum	1.94 (1.46–2.57)	1.50 (0.83–2.72)	1.87 (1.32–2.64)	2.13 (0.93–4.86)	2.63 (1.69–4.09)
Grade	First year (ref.)					
	Second year	1.14 (0.96–1.36)	1.14 (0.80–1.63)	1.09 (0.88–1.34)	1.52 (0.90–2.58)	1.27 (0.94–1.70)
	Third year	1.45 (1.25–1.70)	1.78 (1.32–2.41)	1.39 (1.15–1.67)	0.83 (0.47–1.46)	1.48 (1.14–1.92)
	Fourth year	2.25 (1.34–3.78)	1.62 (0.50–5.21)	2.72 (1.54–4.80)	0.00 (0.00–Inf)	2.37 (1.02–5.51)
Residence on campus	No (ref.)					
	Yes	1.39 (1.19–1.63)	1.44 (1.06–1.95)	1.40 (1.15–1.69)	1.02 (0.62–1.68)	1.39 (1.07–1.81)
Smoking	No (ref.)					
	Yes	1.88 (1.48–2.39)	0.48 (0.21–1.07)	2.20 (1.68–2.88)	3.60 (1.94–6.68)	3.33 (2.42–4.59)
Alcohol consumption	No (ref.)					
	Yes	1.58 (1.33–1.86)	0.72 (0.48–1.10)	1.86 (1.53–2.25)	2.12 (1.26–3.56)	2.30 (1.79–2.96)
Sexual behavior	Never active (ref.)					
	Ever active	2.69 (2.16–3.36)	1.25 (0.73–2.15)	3.05 (2.37–3.92)	2.42 (1.16–5.05)	4.19 (3.09–5.70)
Frequency of sexual behavior in the last month	0 (ref.)					
	1/w	2.03 (1.45–2.84)	1.24 (0.58–2.66)	2.32 (1.58–3.41)	1.81 (0.57–5.77)	2.17 (1.26–3.76)
	2–3/w	1.54 (0.66–3.58)	0.92 (0.13–6.67)	1.57 (0.57–4.33)	0.00 (0.00–Inf)	2.44 (0.76–7.82)
	4–5/w	4.34 (1.42–13.20)	5.61 (1.31–24.08)	4.78 (1.38–16.56)	11.91 (1.56–90.71)	14.40 (4.71–44.03)
	>5/w	6.15 (4.35–8.69)	1.11 (0.35–3.49)	7.78 (5.39–11.21)	5.09 (1.84–14.12)	10.91 (7.18–16.58)
Physical disease	No (ref.)					
	Yes	2.46 (2.03–3.00)	1.82 (1.23–2.72)	2.43 (1.92–3.06)	3.16 (1.77–5.65)	2.46 (1.79–3.37)
Gastropathy	No (ref.)					
	Yes	2.51 (1.62–3.89)	1.49 (0.55–4.06)	2.44 (1.45–4.12)	3.70 (1.16–11.86)	2.34 (1.14–4.81)
Coryza	No (ref.)					
	Yes	0.81 (0.35–1.84)	1.07 (0.26–4.36)	0.81 (0.30–2.22)	1.73 (0.24–12.57)	0.40 (0.06–2.87)
Chronic constipation	No (ref.)					
	Yes	2.78 (2.38–3.26)	1.65 (1.18–2.32)	2.86 (2.37–3.45)	5.01 (3.16–7.93)	2.97 (2.31–3.83)
Game addiction	No (ref.)					
	Yes	1.60 (1.29–1.97)	0.76 (0.44–1.30)	1.89 (1.48–2.41)	1.90 (0.97–3.69)	2.14 (1.56–2.94)
Single parent	No (ref.)					
	Yes	1.31 (1.04–1.66)	1.15 (0.72–1.84)	1.39 (1.05–1.83)	0.96 (0.39–2.37)	1.80 (1.27–2.55)
Father’s education level	Bachelor (ref.)					
	High school & below	1.41 (1.12–1.78)	1.89 (1.15–3.11)	1.39 (1.05–1.84)	0.79 (0.40–1.55)	1.21 (0.83–1.78)
	Junior college	1.21 (0.92–1.61)	1.36 (0.75–2.48)	1.15 (0.81–1.62)	1.23 (0.56–2.73)	1.21 (0.76–1.92)
	Master	1.48 (0.75–2.92)	2.17 (0.63–7.49)	1.55 (0.69–3.46)	0.00 (0.00-Inf)	0.81 (0.19–3.41)
	PhD	3.33 (2.15–5.14)	0.92 (0.21–4.01)	4.17 (2.57–6.76)	2.37 (0.65–8.68)	6.92 (3.97–12.08)
Mother’s education level	Bachelor (ref.)					
	High school & below	1.50 (1.17–1.92)	1.54 (0.96–2.47)	1.51 (1.11–2.05)	0.94 (0.45–1.98)	1.35 (0.89–2.05)
	Junior college	1.45 (1.07–1.96)	0.94 (0.50–1.78)	1.61 (1.11–2.33)	1.46 (0.60–3.54)	1.26 (0.75–2.12)
	Master	2.80 (1.47–5.36)	0.82 (0.11–6.16)	3.24 (1.54–6.82)	1.95 (0.24–15.76)	3.90 (1.56–9.74)
	PhD	3.51 (2.22–5.55)	0.82 (0.19–3.55)	4.70 (2.82–7.84)	1.96 (0.41–9.30)	7.59 (4.19–13.74)
MMHI-60	Normal (ref.)					
	Mild	2.13 (1.86–2.45)	1.51 (1.17–1.96)	2.18 (1.84–2.58)	4.43 (2.58–7.60)	2.12 (1.67–2.70)
	Moderate	4.09 (3.26–5.14)	2.11 (1.31–3.41)	4.49 (3.45–5.86)	5.60 (2.43–12.93)	4.74 (3.32–6.78)
	Serious and very serious	10.32 (5.54–19.22)	0.00 (0.00-Inf)	10.67 (5.37–21.22)	33.14 (9.42–116.57)	30.83 (16.08–59.11)

Abbreviations: UI, urinary incontinence; BMI, body mass index; MMHI-60, Mental Health Inventory of Middle-School Students; NE, nocturnal enuresis; CI: confidence interval; ref.: reference; Inf: infinity.

**Table 5:** Multivariable analysis of factors associated with UI and subtypes.

Related Factors		UI OR (95%CI)	Stress UI OR (95%CI)	Urgency UI OR (95%CI)	Mixed UI OR (95%CI)	NE OR (95%CI)
Age (years)	14–16 (ref.)					
	17–20	-	-	-	0.48 (0.29–0.80)	-
Sex	Boys (ref.)					
	Girls	1.27 (1.11–1.45)	3.66 (2.70–4.95)	-	-	-
Inhabitation	Urban (ref.)					
	Rural	1.37 (1.17–1.60)	1.21 (0.90–1.62)	1.43 (1.20–1.70)	-	1.74 (1.33–2.28)
	Rural-urban continuum	1.45 (1.08–1.95)	1.28 (0.70–2.33)	1.37 (0.95–1.96)	-	1.89 (1.19–3.02)
Grade	First year (ref.)					
	Second year	1.18 (0.98–1.40)	1.17 (0.82–1.67)	1.12 (0.90–1.39)	1.95 (1.12–3.38)	1.36 (1.01–1.84)
	Third year	1.36 (1.16–1.59)	1.66 (1.23–2.25)	1.29 (1.06–1.56)	1.08 (0.57–2.07)	1.39 (1.06–1.82)
	Fourth year	1.51 (0.87–2.65)	1.57 (0.48–5.13)	1.86 (1.02–3.41)	0.00 (0.00–Inf)	1.05 (0.39–2.78)
Smoking	No (ref.)					
	Yes	-	-	-	2.24 (1.15–4.35)	-
Alcohol consumption	No (ref.)					
	Yes	-	-	1.27 (1.03–1.57)	-	1.43 (1.08–1.89)
Frequency of sexual behavior in the last month	0 (ref.)					
	1/w	1.82 (1.28–2.59)	1.52 (0.70–3.28)	1.86 (1.25–2.78)	-	1.52 (0.85–2.72)
	2–3/w	2.96 (1.03–8.54)	1.45 (0.20–10.71)	1.19 (0.42–3.34)	-	1.85 (0.57–6.04)
	4–5/w	2.07 (0.58–7.41)	7.71 (1.65–35.90)	2.36 (0.67–8.27)	-	4.54 (1.08–19.03)
	>5/w	4.48 (3.07–6.54)	1.54 (0.47–5.02)	5.16 (3.48–7.66)	-	5.73 (3.54–9.29)
Physical disease	No (ref.)					
	Yes	1.73 (1.40–2.13)	1.61 (1.06–2.44)	1.59 (1.24–2.04)	1.86 (1.00–3.44)	1.44 (1.02–2.05)
Chronic constipation	No (ref.)					
	Yes	2.03 (1.72–2.40)	1.30 (0.92–1.85)	2.06 (1.69–2.51)	3.64 (2.25–5.90)	1.93 (1.47–2.55)
Father’s education level	Bachelor (ref.)					
	High school & below	1.28 (1.00–1.64)	1.76 (1.05–2.96)	-	-	0.98 (0.65–1.48)
	Junior college	1.26 (0.94–1.68)	1.41 (0.77–2.58)	-	-	1.20 (0.75–1.93)
	Master	1.39 (0.69–2.81)	2.43 (0.69–8.56)	-	-	0.69 (0.16–2.96)
	PhD	2.02 (1.24–3.29)	0.95 (0.21–4.32)	-	-	2.97 (1.56–5.62)
MMHI-60	Normal (ref.)					
	Mild	1.88 (1.63–2.16)	1.43 (1.10–1.86)	1.88 (1.58–2.23)	3.69 (2.14–6.38)	1.80 (1.41–2.30)
	Moderate	2.87 (2.25–3.65)	1.82 (1.10–3.00)	3.01 (2.27–3.98)	3.55 (1.49–8.45)	2.94 (2.00–4.32)
	Serious and very serious	4.57 (2.27–9.27)	0.00 (0.00-Inf)	4.40 (2.03–9.53)	12.79 (3.23–50.61)	11.57 (5.35–25.03)

Abbreviations: UI, urinary incontinence; MMHI-60, Mental Health Inventory of Middle-School Students; NE: nocturnal enuresis; CI: confidence interval; ref.: reference; Inf: infinity.
